# Counterfactual explanation-based targeted regulation of key chemical indicators for improving tobacco leaf sensory quality

**DOI:** 10.3389/fpls.2026.1991648

**Published:** 2026-10-22

**Authors:** Hexin Chen, Wenting Li, Junwei Guo, Hongbo Wang, Song Yang, Ran Wan, Yanling Liu, Zhenyu Liu, Guoxin Qin, Cong Wang, Zeli Wang, Haiyue Su, Xianyue Zhu, Zhen Wang, Zhicai Wang, Cong Nie, Le Zhao, Shan Li, Xufeng Wang

**Affiliations:** 1 Zhengzhou Tobacco Research Institute of CNTC, Zhengzhou, China; 2 Technology R&D Center, Gansu Tobacco Industrial Co., Ltd., Lanzhou, China; 3 Technology Center, China Tobacco Shaanxi Industrial Co., Ltd., Xi’an, China

**Keywords:** chemical indicators, counterfactual explanations, decision models, targeted regulation, tobacco leaf sensory quality

## Abstract

**Introduction:**

To address the need for the value-added utilization of tobacco leaves with low sensory quality, this study proposed a targeted chemical composition regulation method based on counterfactual explanations and preliminarily evaluated the resulting chemical adjustment strategies using samples processed by a rotary-drum tobacco high-temperature (THT) drying process.

**Methods:**

Based on 70 chemical indicators and sensory smoking evaluation data from 354 tobacco leaf samples, binary shrinkage linear discriminant analysis (shrinkage LDA) models were developed to distinguish between the high- and low-sensory-quality groups in terms of aroma quality, aroma intensity, and offensive odor. Incremental feature selection (IFS) was employed to identify key chemical indicators, after which indicators with low adjustability under THT conditions were further excluded. The decision models for counterfactual explanations were then reconstructed using the remaining indicators. Sequential least squares programming (SLSQP) was applied to generate chemical-indicator adjustment pathways for improving sensory quality, and the resulting pathways were validated through a THT experiment conducted at 215 °C.

**Results:**

The results showed that: (1) following incremental feature selection and further screening based on the adjustability of chemical indicators under THT conditions, 11, 9, and 10 key chemical indicators were ultimately retained in the decision models for aroma quality, aroma intensity, and offensive odor, respectively; (2) all the developed models achieved classification accuracies above 84% on the test sets, indicating good mapping relationships between the key chemical indicators and sensory quality; (3) the directional agreement between the theoretically predicted adjustments and the experimentally measured changes ranged from 66.7% to 72.7%. Moreover, indicators showing directional agreement contributed significantly more to improvements in discriminant scores than those showing opposite directions, confirming their dominant role in sensory quality improvement.

**Discussion:**

These findings demonstrate that counterfactual explanations can effectively predict optimization pathways in complex chemical systems, thereby providing theoretical support and methodological guidance for the targeted improvement of tobacco leaves with low sensory quality.

## 1 Introduction

Tobacco leaves with low sensory quality may exhibit one or more quality defects, such as coarse aroma quality, insufficient aroma intensity, pronounced green and woody offensive odor, strong irritation, inappropriate strength, an unclean aftertaste, bitterness, and astringency. Consequently, these tobacco leaves cannot directly meet the formulation requirements of modern cigarette products. They frequently accumulate in large quantities in industrial inventories, imposing a substantial economic burden on cigarette manufacturers (Zhu, 2010). Because tobacco leaf quality and sensory characteristics are influenced by multiple factors, with chemical composition being one of the major factors (Xiong et al., 2023), regulating the chemical composition of tobacco leaves through biological treatments, high-temperature processing, and other technological approaches has become an important means of improving tobacco leaf quality (Hao et al., 2022; An et al., 2012). Interventions such as microbial fermentation, enzymatic catalysis, and combined enzyme-microorganism treatments can target specific macromolecular constituents in tobacco leaves, including starch, pectin, and proteins. These treatments degrade macromolecules to mitigate undesirable sensory characteristics and generate compounds, such as aldehydes, ketones, alcohols, nonvolatile organic acids, and fatty acids, that may contribute to aroma and the overall sensory profile, thereby improving the suitability of tobacco leaves with low sensory quality for use in cigarette blends (Liu et al., 2024; Su et al., 2025). High-temperature roasting, as a commonly used dry-heat processing method, regulates the chemical composition, fiber structure, and thermal conversion characteristics of tobacco leaves through controlled thermal reactions (Cheng et al., 2026). It can alter the contents of total sugars, reducing sugars, nicotine, total nitrogen, organic acids, and volatile aroma compounds, as well as chemical indices such as the sugar-to-nicotine and nitrogen-to-nicotine ratios. These changes improve the balance among chemical constituents, reduce undesirable and irritating smoke characteristics, enhance processing performance, and ultimately increase the industrial usability of different types of tobacco materials (Liu et al., 2016; Liang et al., 2025).

During combustion and smoking, chemical substances in tobacco leaves undergo a series of complex thermochemical reactions, including pyrolysis, thermal synthesis, redox reactions, and polymerization, thereby shaping core sensory attributes such as aroma quality, aroma intensity, and offensive odor (Wang, 2018). Thus, the sensory quality of tobacco leaves is not determined directly by the concentration of a single constituent but instead results from the synergistic effects and dynamic balance of multiple chemical constituents. Elucidating the mapping relationships between chemical indicators and sensory quality is therefore an important approach to improving tobacco leaf quality. For example, the taste characteristics of tobacco smoke are largely influenced by the balance and coordination between acidic pyrolysis products derived from water-soluble sugars and alkaline pyrolysis products derived from nitrogen-containing compounds during tobacco combustion (Xiao, 1997). Some aroma compounds impart pleasant characteristic aromas at trace levels; however, once the original chemical balance is disrupted, excessively high concentrations may instead produce offensive odor or intensify sensory irritation (Shi, 2011). Previous studies have employed partial least squares regression, multivariate statistical analysis, and extreme gradient boosting combined with the SHAP interpretation framework to reveal relationships between coordination indices, such as the sugar-to-nicotine and sugar-to-nitrogen ratios, and tobacco sensory quality. These approaches have also identified key indicators affecting sensory quality from multiple categories of chemical constituents, including sugars, alkaloids, organic acids, and inorganic ions. However, these studies have primarily focused on the associations between chemical indicators and sensory quality and on the contributions of individual indicators, without further determining the specific directions and magnitudes required to adjust individual indicators and their combinations (Lin and Zhang, 2016; Zhou et al., 2024; Bie et al., 2022).

Wachter proposed the theory of counterfactual explanations as an approach to explainable artificial intelligence (AI) (Wachter et al., 2018). Based on an established decision model, this method makes the smallest possible modifications to input features to shift the model prediction from its original state to a desired state. It thereby reveals which features must be adjusted, together with the required magnitudes of adjustment, and provides an intuitive explanation of individual model predictions (Guidotti, 2024; Verma et al., 2024). In recent years, counterfactual explanations have demonstrated considerable potential for targeted optimization in fields such as drug molecular design and engineering operations research. In molecular design, relevant studies have employed local mutations or generative substitutions of molecular structures to identify the key structural features and modification strategies capable of inducing desired changes in predicted biological activity or chemical properties, thereby providing actionable guidance for targeted molecular design (Wellawatte et al., 2022; Janisiów et al., 2026). In engineering operations research, counterfactual generation mechanisms have been combined with methods such as inverse constraint programming to obtain parameter adjustment strategies with relatively low modification costs while satisfying desired objectives (Korikov and Beck, 2021). Collectively, these studies demonstrate that counterfactual explanations can generate adjustment strategies under predefined target states and constraints, providing a methodological basis for inversely deriving the directions and magnitudes of chemical-indicator adjustments required to improve the sensory quality of tobacco leaves.

Therefore, based on binary classification models of tobacco sensory quality, this study employed counterfactual explanations to identify minimal chemical-adjustment pathways for improving tobacco sensory quality under reasonable constraints. Process-treatment experiments were subsequently conducted to evaluate and validate the effectiveness of the resulting regulation strategies. This approach provides a new quantitative interpretation tool for elucidating the intrinsic relationships between tobacco chemical indicators and sensory quality while offering practical guidance for industrial enterprises seeking to improve the quality of tobacco leaves with low sensory quality through targeted interventions.

## 2 Materials and methods

### 2.1 Materials and instruments

Modeling samples: A total of 354 single-grade flue-cured tobacco leaf samples were collected from the Yunnan production region of China and used to construct and train the binary classification models of tobacco leaf sensory quality.

Validation samples: Three tobacco leaf samples with low sensory quality were collected as the control group (YS group). The corresponding samples subjected to the rotary-drum tobacco high-temperature (THT) drying process at 215 °C were used as the experimental group (CL group). Eleven sensory panelists used the triangle test to compare each sample in the YS group with its corresponding sample in the CL group. They assessed whether THT treatment resulted in significant differences in aroma quality, aroma intensity, and offensive odor, and further classified the direction of each change as improvement, deterioration, or no noticeable change. At α= 0.05, a difference between the pre- and post-treatment samples was considered significant when seven or more panelists correctly identified the odd sample. This assessment was used to identify samples whose sensory attributes improved significantly after THT treatment. The observed changes in chemical indicators in these samples were then compared with the corresponding quality-improvement strategies generated by the counterfactual model to preliminarily examine the consistency between the model-generated strategies and the actual treatment results. The evaluation results are presented in **Table 1**. As shown in **Table 1**, THT improved the aroma quality and aroma intensity of samples YS-1 and YS-3 and significantly reduced the offensive odor in sample YS-2. For the remaining sensory attributes, the number of correct identifications did not reach the critical value; therefore, reliable changes in sensory quality before and after treatment could not be established.

**Table 1:** Sensory evaluation results for the control and THT-treated samples.

Sample pair	Panelists (n)	Critical value (α=0.05)	Aroma quality		Aroma intensity		Offensive odor	
			Correct identifications (n)	Change	Correct identifications (n)	Change	Correct identifications (n)	Change
YS-1/CL-1	11	7	9	Improved	10	Improved	4	No evident change
YS-2/CL-2	11	7	4	No evident change	5	No evident change	8	Improved
YS-3/CL-3	11	7	8	Improved	7	Improved	3	No evident change

Instruments: Antaris II Fourier Transform Near-Infrared Spectrometer (equipped with an integrating sphere diffuse reflectance sampling system, Thermo Scientific, USA); LC-2232 Adjustable Temperature Oven (Espec Environmental Equipment Co., Ltd., Shanghai, China); ZM200 grinder (Retsch, Germany); BSA124S Analytical Balance (readability: 0.1 mg, Sartorius, Germany); ZJ118 Cigarette-Making Machine (Changde Tobacco Machinery Co., Ltd., China); EVO Tobacco Cutter (Garbuio, Italy).

### 2.2 Methods

#### 2.2.1 Sample pretreatment

All tobacco leaf samples were dried in accordance with the tobacco industry standard YC/T 31-1996, ground using a grinder fitted with a 0.250 mm (60-mesh) sieve, thoroughly homogenized, and stored in sealed bags.

#### 2.2.2 Near-infrared spectral acquisition

Environmental conditions: The ambient temperature was maintained at 20~27°C, and the relative humidity was maintained at 30%~50%.

Near-infrared spectral acquisition parameters: Spectra were acquired over a wavenumber range of 4000~10000 cm^-1^, with 64 scans per measurement and a spectral resolution of 8 cm^-1.^

Spectral acquisition: Background spectra were collected every 20 min. Two spectra were acquired for each sample, and the correlation coefficient between the two spectra was required to exceed 0.9999.

Spectral preprocessing: Multiplicative scatter correction (MSC) was applied to reduce scattering effects caused by variations in sample particle size and uneven particle distribution. Savitzky-Golay convolution smoothing, with a window length of 17 and a polynomial order of 2, was used to reduce high-frequency random noise and improve the signal-to-noise ratio. A first-derivative transformation was subsequently applied to correct spectral baseline shifts.

#### 2.2.3 Acquisition of chemical indicator data

The near-infrared analysis platform of China National Tobacco Corporation was used to predict 70 major and semi-micro chemical indicators related to tobacco leaf quality and style (Guo et al., 2023; Li et al., 2025; Liang et al., 2022), including routine chemical components, cations and anions, polyphenols, polyacids and higher fatty acids, amino acids, Amadori compounds, and other components. The specific chemical indicators are listed in **Table 2**.

**Table 2:** Classification of chemical indicators in tobacco leaves.

No.	Type	Specific feature	Number of chemical indicators
1	Routine chemical indicators	Total alkaloids, Reducing sugar, Total sugar, Total nitrogen, Starch	5
2	Cations and anions	Potassium, Chlorine, Sulfate, Phosphate, Magnesium, Calcium	6
3	Polyphenols	Neochlorogenic acid, Chlorogenic acid, Cryptochlorogenic acid, Scopoletin, Rutin	5
4	Polyacids and higher fatty acids	Oxalic acid, Malonic acid, Succinic acid, Malic acid, Citric acid, Vanillic acid, Myristic acid, Palmitic acid, Linoleic acid, Oleic acid + Linolenic acid, Stearic acid, Arachidic acid	12
5	Amino acids	Aspartic acid (Asp), Threonine (Thr), Serine (Ser), Asparagine (Asn), Glutamic acid(Glu), Glutamine(Gln), Glycine(Gly), Alanine(Ala), Valine(Val), Cystine(Cys), Methionine(Met), Isoleucine(Ile), Leucine(Leu), Tyrosine(Tyr), Phenylalanine(Phe), 4-Aminobutyric acid (GABA), Lysine(Lys), Histidine(His), Tryptophan(Trp), Arginine(Arg), Proline(Pro)	21
6	Amadori compounds	N-(1-Deoxy-d-glucose-1-yl) Ammonia (Glu-An), N-(1-deoxy-D-fructos-1-yl) aminobutyric (Fru-Amb), N-(1-deoxy-D-fructos-1-yl) Histidine (Fru-His), N-(1-deoxy-D-fructos-1-yl) Proline (Fru-Pro), N-(1-deoxy-D-fructos-1-yl) Valine (Fru-Val), N-(1-deoxy-D-fructos-1-yl) Threonine (Fru-Thr), N-(1-deoxy-D-fructos-1-yl) Glycine (Fru-Gly), N-(1-deoxy-D-fructos-1-yl) Alanine (Fru-Ala), N-(1-deoxy-D-fructos-1-yl) Asparagine (Fru-Asn), N-(1-deoxy-D-fructos-1-yl) Aspartic acid (Fru-Asp), N-(1-deoxy-D-fructos-1-yl) Glutamine (Fru-Gln), N-(1-deoxy-D-fructos-1-yl) Glutamic acid (Fru-Glu), N-(1-deoxy-D-fructos-1-yl) Isoleucine (Fru-Ile), N-(1-deoxy-D-fructos-1-yl) Leucine (Fru-Leu), N-(1-deoxy-D-fructos-1-yl) Tyrosine (Fru-Tyr), N-(1-deoxy-D-fructos-1-yl) Phenylalanine (Fru-Phe), N-(1-deoxy-D-fructos-1-yl) Tryptophan (Fru-Trp)	17
7	Others	pH, Dichloromethane extract, Solanesol, Neophytadiene	4
Total			70

#### 2.2.4 Acquisition of sensory quality scores

All single-grade tobacco leaves were processed online in the tobacco-processing workshop. The cut width was 1.0 mm, and the target moisture content was controlled at 12.0%. Cigarettes were then manufactured using standard auxiliary materials for a Class II cigarette from Lanzhou on a ZJ118 cigarette-making machine. The auxiliary materials included CTM VGB 60–37 SL15 horizontal-ribbed cigarette paper, 72-mm double-row perforated tipping paper, and 120-mm high-permeability plug-wrap paper filters.

After manufacture, the cigarettes were screened to remove abnormal ones. The selection criteria were a cigarette mass of 880 ± 20 mg, a draw resistance of 1,080 ± 100 Pa, and total ventilation of 20% ± 4%. Before sensory assessment, the cigarettes were conditioned for at least 48 h at 22 ± 1 °C and 65% relative humidity to ensure uniform moisture content. Aroma quality, aroma intensity, and offensive odor were then assessed with reference to tobacco industry standard YC/T 138-1998. The results are presented in **Table 3**.

**Table 3:** Sensory evaluation scoring criteria.

Aroma quality (15)	Aroma intensity (15)	Offensive odor (9)
Good (12-15)	Abundant (12-15)	Absent (8-9)
Fairly good (9-12)	Fairly abundant (9-12)	Negligible (7-8)
Moderately good (7-9)	Moderately abundant (7-9)	Very slight (6-7)
Above average (5-7)	Substantial (5-7)	Barely perceptible (5-6)
Average (4-5)	Fairly substantial (4-5)	Slightly perceptible (4-5)
Below average (3-4)	Moderately substantial (3-4)	Perceptible (3-4)
Slightly poor (2-3)	Present (2-3)	Moderately strong (2-3)
Fairly poor (1-2)	Slight (1-2)	Strong (1-2)
Poor (0-1)	Low (0-1)	Very strong (0-1)

#### 2.2.5 Tobacco leaf dataset partitioning

Classification of Tobacco Leaves into High- and Low-Sensory-Quality Groups: The upper and lower quartiles of the sensory quality scores were used as classification thresholds. Samples with sensory scores greater than or equal to the upper quartile were assigned to the high-sensory-quality group for the corresponding sensory attribute, whereas samples with scores less than or equal to the lower quartile were assigned to the low-sensory-quality group. Samples within the intermediate ambiguous range were excluded. For example, when modeling aroma quality, the dataset comprised a high-aroma-quality tobacco leaf dataset and a low-aroma-quality tobacco leaf dataset, each containing the aroma quality scores and 70 chemical indicators.

Outlier removal: Outliers in the two tobacco leaf datasets were identified and removed using the median absolute deviation (MAD) method. First, Z-score standardization was applied to each chemical indicator to eliminate differences in measurement units and numerical scales among the indicators. For each sample, the Euclidean distance from the sample to the mean centroid of its assigned class was calculated using the standardized chemical indicators. The MAD of the Euclidean distances within each class was then calculated as the median of the absolute deviations of all sample distances from the median distance of that class. Three times the MAD was used as the threshold for outlier removal. A sample was identified as an outlier and removed when the absolute deviation of its Euclidean distance from the class median distance exceeded this threshold.

Dataset for counterfactual explanation modeling: After outlier removal, stratified sampling was used to divide the tobacco leaf dataset into a training set and a test set at a ratio of 7:3. The training set was used to rank the contributions of the chemical indicators, select the key chemical indicators, and construct the final classification models, whereas the test set was used to evaluate the performance of the final models.

#### 2.2.6 Counterfactual explanation method for sensory quality improvement

First, binary classification models of tobacco leaf sensory quality were constructed using all chemical indicators within the training set. Stratified five-fold cross-validation was performed to obtain the feature-weight coefficients of the model decision functions, and the contribution ranking of the chemical indicators was determined according to their mean absolute weights. Incremental feature selection was then applied to identify the key chemical indicators. These indicators were further screened according to their adjustability under the THT process to improve the practical feasibility of the counterfactual adjustment strategies. Binary classification models of sensory quality were subsequently reconstructed using the remaining key chemical indicators and used as the decision models underlying the counterfactual explanation method. Finally, the decision models were used to optimize adjustments to the chemical indicators and determine the resulting class predictions, thereby generating chemical-indicator adjustment strategies for improving tobacco leaf sensory quality.

##### 2.2.6.1 Binary classification models of tobacco leaf sensory quality

###### 2.2.6.1.1 Selection of key chemical indicators for the binary classification models of tobacco leaf sensory quality

Feature dimensionality reduction was performed through feature importance evaluation combined with incremental feature selection (IFS) (Chen et al., 2020) to identify the key chemical indicators influencing the classification decisions for each sensory attribute.

1. Binary classification models were constructed separately for each sensory attribute using shrinkage linear discriminant analysis (shrinkage LDA) (Schäfer and Strimmer, 2005; Blankertz et al., 2011). Five-fold cross-validation was performed within the training set to evaluate the classification performance and stability of each sensory classification model. The feature-weight coefficients of the model decision function were extracted from each cross-validation fold, and the mean absolute weight coefficient was calculated for each chemical indicator. This mean value was used to quantify the contribution of each chemical indicator to the classification of the corresponding sensory attribute, and the indicators were ranked in descending order of contribution.

2. Incremental feature selection (IFS) was performed by sequentially introducing chemical indicators in descending order of their contributions. Feature subsets containing different numbers of chemical indicators were constructed, and a shrinkage linear discriminant analysis (shrinkage LDA) binary classification model for the corresponding sensory attribute was reconstructed using each feature subset. Five-fold cross-validation within the training set was used to calculate the mean area under the receiver operating characteristic curve (AUC) for each candidate model and to evaluate changes in classification performance as chemical indicators were progressively introduced. Candidate models with a five-fold mean AUC within 0.01 of the highest five-fold mean AUC were considered to satisfy the selection criterion. Among these models, the model containing the fewest chemical indicators was selected, and its included indicators were defined as the key chemical indicators for the corresponding sensory attribute. The identified key chemical indicators were further screened based on their adjustability under THT processing conditions, and the remaining key chemical indicators were used to construct the subsequent decision models for counterfactual explanations.

###### 2.2.6.1.2 Construction and evaluation of decision models for counterfactual explanations

The binary classification models of tobacco sensory quality for the respective sensory attributes were reconstructed using the selected key chemical indicators and subsequently used as the decision models for counterfactual explanations aimed at improving each sensory attribute (Ribeiro et al., 2016). The classification performance of the decision models was comprehensively evaluated using accuracy (A), precision (P), recall (R), and the F1 score (F1). Values closer to 1 indicate better classification performance.

(1) Accuracy. Accuracy represents the proportion of samples correctly predicted by the classification model relative to the total number of samples, (N); that is, the proportion of true-positive and true-negative samples combined relative to the total number of samples. Accuracy was calculated using Equation 1:

(1) $$Accuracy=\frac{TP+TN}{TP+TN+FP+FN}$$

(2) Precision. Precision represents the proportion of samples that are actually positive among all samples predicted as positive by the classification model. Precision was calculated using Equation 2:

(2) $$Precision=\frac{TP}{TP+FP}$$

(3) Recall. Recall represents the proportion of samples correctly predicted as positive by the classification model among all samples that are actually positive. Recall was calculated using Equation 3:

(3) $$Recall=\frac{TP}{TP+FN}$$

(4) F1 Score. The F1 score is the harmonic mean of precision and recall and was calculated using Equation 4:

(4) $$F1-Score=2\times \frac{Precision\times Recall}{Precision+Recall}$$

where (TP) (true positive) denotes the number of samples that actually belong to the positive class and are correctly predicted as positive; (TN) (true negative) denotes the number of samples that actually belong to the negative class and are correctly predicted as negative; (FP) (false positive) denotes the number of samples that actually belong to the negative class but are incorrectly predicted as positive; (FN) (false negative) denotes the number of samples that actually belong to the positive class but are incorrectly predicted as negative; (N) denotes the total number of samples in the test set, where (N=TP+TN+FP+FN); and (A), (P), (R), and (F1) denote accuracy, precision, recall, and the F1 score, respectively.

Good discriminative performance of the decision models indicates a strong relationship between the chemical indicators included in the models and the corresponding sensory attributes. This suggests that targeted adjustment of these indicators could shift the chemical profiles of low-sensory-quality tobacco leaf samples toward the profile associated with the high-sensory-quality group, potentially providing an approach to improving their sensory characteristics.

##### 2.2.6.2 Sensory quality improvement through targeted optimization of chemical indicators

A constraint- and gradient-based continuous numerical optimization framework for minimum-perturbation search was employed (Nie, 2025). By minimizing perturbations to the chemical indicators as the optimization cost (Mothilal et al., 2020), theoretical chemical-indicator adjustment strategies for sensory quality improvement were derived, and the optimized samples were determined. The procedure was performed as follows:

###### 2.2.6.2.1 Determination of the targeted optimization starting points and objective function for tobacco leaf samples with low sensory quality

The original chemical-indicator vector *x*_0_ of the tobacco leaf sample with low sensory quality to be improved was used as the starting point. The chemical indicators of the optimized sample were denoted by *x*′, and the chemical-indicator modification vector was defined as δ^*^ = *x*′ − *x*_0_. Minimization of the norm of the chemical-indicator modification vector, ( $\frac{1}{2}{\left\|{\text{δ}}^{*}\right\|}_{2}^{2}$), was used as the objective function.

###### 2.2.6.2.2 Iterative optimization using the SLSQP algorithm

The gradient-based sequential least squares programming (SLSQP) algorithm (Ma et al., 2024) was used to derive optimized samples corresponding to the tobacco leaf samples with low sensory quality. The objective function and constraints are given in Equations 5 and 6, respectively.

(5) $$\underset{\text{d}}{\min}\left[\nabla f{\left({\text{x}}^{\text{(t)}}\right)}^{⊤}{\text{d}}^{*}+\frac{1}{2}{\text{d}}^{*⊤}{\text{B}}^{\left(\text{t}\right)}{\text{d}}^{*}\right]$$

(6) $$s.t.\;L_{t}\leq A_{t}d^{*}\leq U_{t}$$

where d^*^ denotes the candidate update vector to be solved; *B*^(t)^ denotes the Hessian matrix of the objective function, namely, the norm of the chemical-indicator modification vector; *A*_t_ denotes the constraint matrix obtained by linearizing the hard boundary constraints at the current point; and *L*_t_ and *U*_t_ denote the corresponding linearized lower and upper bounds, respectively. These constraints require each chemical indicator of the optimized sample *x’* to remain within the corresponding minimum-maximum range of that indicator in the training set.

After d^*^ was obtained, a line search was performed along the direction of d^*^ to determine the optimal step size α^*^. The initial step size was set to *α* = 1 and was iteratively halved according to *α*→0.5*α*. A step size that satisfied the hard boundary constraints and increased the discriminant score of the decision model was selected as the optimal step size α^*^. Accordingly, the resulting chemical-indicator perturbation vector was defined as shown in Equation 7:

(7) $$\delta^{\left(\text{t}\right)}={\text{α}}^{*}\cdot {\text{d}}^{*}$$

The virtual sample *x*^(t)^ was updated to obtain the new virtual sample *x*^(t+1)^, as shown in Equation 8:

(8) $$x^{\left(\text{t}+1\right)}=x^{\left(\text{t}\right)}+\delta^{\left(\text{t}\right)}$$

The iterative procedure described above was repeated until the chemical indicators of the virtual sample *x*^(t+1)^ satisfied the hard boundary constraints and its decision-model discriminant score was greater than or equal to the discriminant-score centroid of the high-sensory-quality group *μ*_high_, defined as the mean model discriminant score of all correctly classified high-sensory-quality samples in the training set. The resulting virtual sample was then regarded as the optimized sample *x*′ corresponding to the original tobacco leaf sample with low sensory quality.

#### 2.2.7 Data processing methods

All data analysis, model construction and visualization procedures were implemented in Python 3.9, with open-source toolkits including Scikit-learn and Matplotlib employed for data analysis.

## 3 Results and discussion

### 3.1 Tobacco leaf dataset screening

To ensure that the classified tobacco leaf datasets contained sufficient numbers of samples and exhibited clear quality differences between classes, the upper and lower quartiles of the sensory-score distributions were used to define reasonable classification thresholds for the high- and low-sensory-quality groups.

The boxplot distributions of the sensory quality scores for aroma quality, aroma intensity, and offensive odor are shown in **Figure 1**. The upper-quartile scores of 10.38, 10.625, and 7.25 were used as the screening thresholds for the high-sensory-quality groups characterized by high aroma quality, high aroma intensity, and low offensive odor, respectively. The lower-quartile scores of 8.38, 8.45, and 6.50 were used as the screening thresholds for the low-sensory-quality groups characterized by low aroma quality, low aroma intensity, and high offensive odor, respectively.

**Figure 1:**
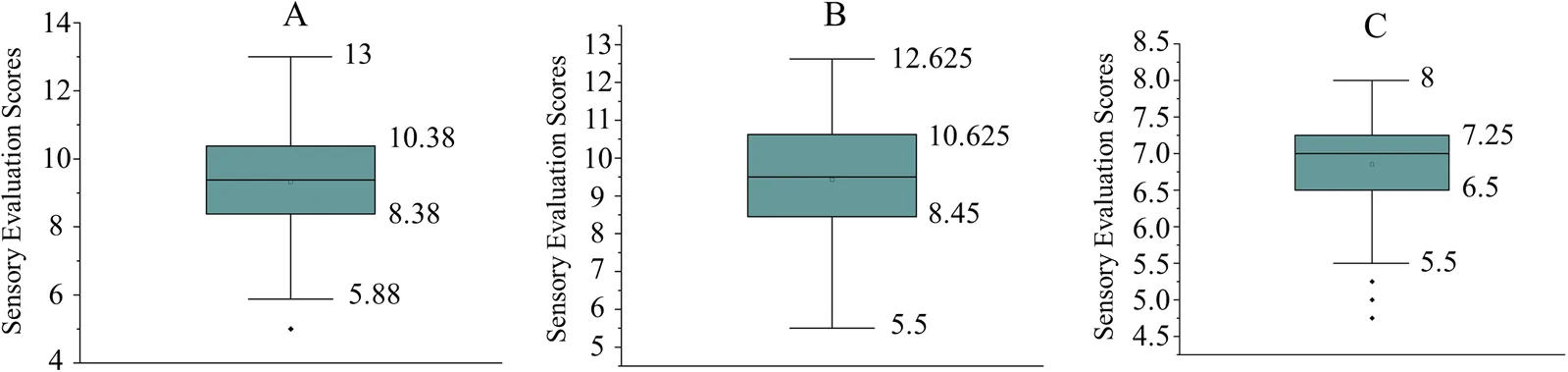
Boxplots of sensory evaluation scores for tobacco leaf samples: **(A)** aroma quality, **(B)** aroma intensity, and **(C)** offensive odor.

The chemical indicators in the high- and low-sensory-quality tobacco leaf datasets for each sensory attribute were subjected to MAD-based outlier detection, and the identified outlier samples were removed. The final sample composition of the dataset for each sensory attribute is presented in **Table 4**. A total of 170, 174, and 185 valid samples remained for aroma quality, aroma intensity, and offensive odor, respectively. Of these, 78, 85, and 85 samples were classified as high-sensory-quality samples, whereas the corresponding numbers of low-sensory-quality samples were 92, 89, and 100, respectively.

**Table 4:** Sample size distributions for aroma quality, aroma intensity, and offensive odor.

Sensory indicator	Retained samples			Excluded samples		
	Total	High sensory quality	Low sensory quality	Ambiguous interval	High sensory quality	Low sensory quality
Aroma quality	170	78	92	175	7	2
Aroma intensity	174	85	89	173	7	0
Offensive odor	185	85	100	146	21	2

High-sensory-quality outliers.

### 3.2 Selection of key chemical indicators for the binary classification models of tobacco leaf sensory quality

Binary classification models of tobacco leaf sensory quality were constructed separately for each sensory attribute, and five-fold cross-validation was used to evaluate their generalization performance and robustness. The accuracy obtained in each fold and the corresponding mean accuracy are presented in **Table 5**. As shown in **Table 5**, the binary classification models for aroma quality and aroma intensity achieved mean accuracies of 90.7% and 87.4%, respectively, indicating strong discriminative performance. The model for offensive odor achieved a mean predictive accuracy of 82.2%, indicating good discriminative performance. For each sensory attribute, the five-fold accuracies remained relatively high and exhibited only small variations, indicating that the classification models were relatively stable and that their classification decision boundaries were reasonably well defined. Therefore, the feature-weight coefficients in the corresponding decision functions could be used to quantify the relative contributions of the chemical indicators to the classification decisions of the respective models.

**Table 5:** Five-fold cross-validation accuracies of the binary classification models for aroma quality, aroma intensity, and offensive odor.

Sensory indicator	Fold 1	Sensory indicator	Fold 2	Fold 3	Fold 4	Fold 5	Mean accuracy
Aroma quality	83.3%	94.1%	85.0%	94.1%	97.1%	90.7%
Aroma intensity	82.9%	97.1%	88.6%	80.0%	88.2%	87.4%
Offensive odor	70.3%	83.8%	94.6%	83.8%	78.4%	82.2%

Using aroma quality as an example, the classification contributions of the chemical indicators are shown in **Figure 2**. Seven indicators, including Ca, vanillic acid, Mg, malic acid, chlorogenic acid, dichloromethane extract, and phosphate, had contribution values greater than 1.00. Ca showed the highest contribution value of 1.65, followed by vanillic acid and Mg. The contribution values of Fru-Tyr, isoleucine, and palmitic acid ranged from 0.75 to 1.00, whereas those of the remaining 60 indicators, including Glu-An, chlorine, and succinic acid, were below 0.75.

**Figure 2:**
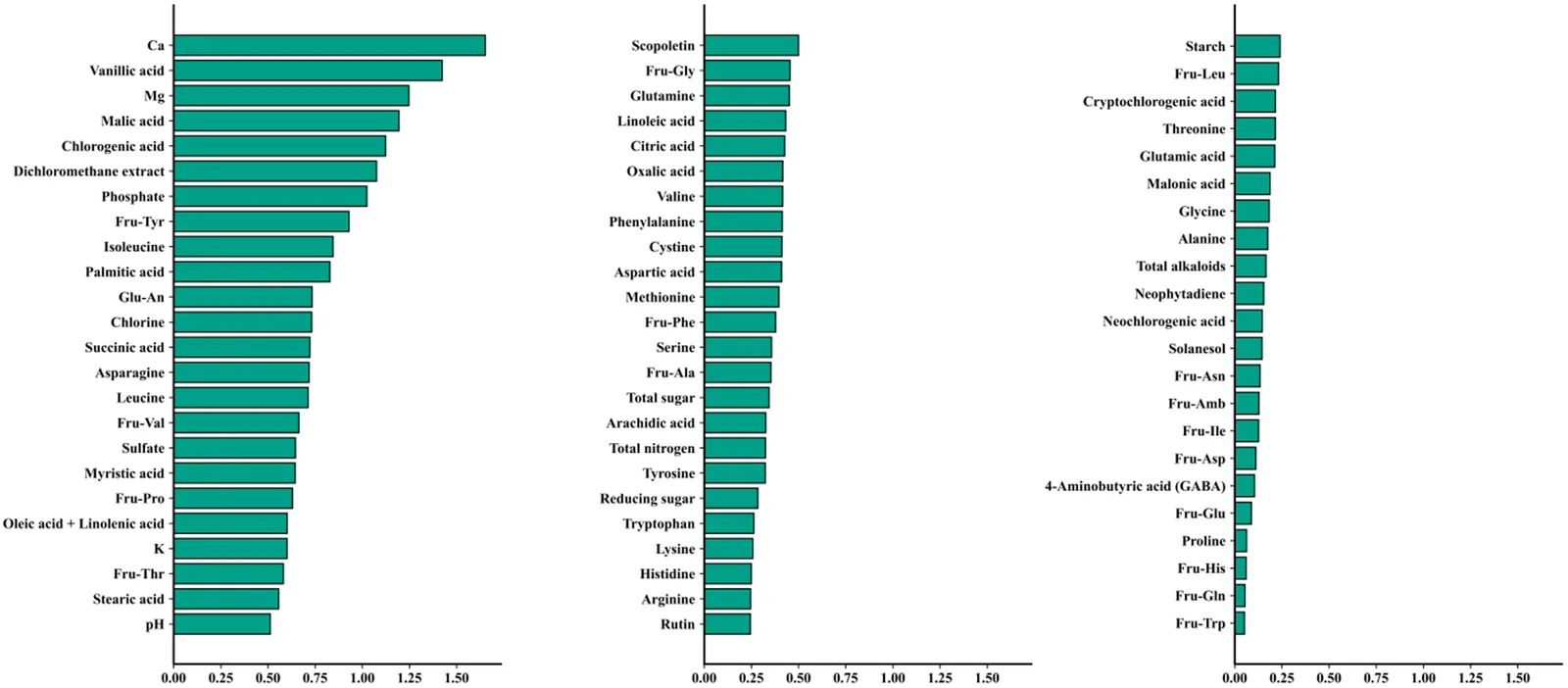
Ranking of chemical indicator contributions in the binary classification model for aroma quality.

Chemical indicators were sequentially introduced according to their contribution rankings for each sensory attribute, and the binary classification models of aroma quality, aroma intensity, and offensive odor were retrained. Changes in the five-fold mean AUC with increasing numbers of chemical indicators are shown in **Figure 3**. As shown in **Figure 3**, the five-fold mean AUC values of the binary classification models for aroma quality, aroma intensity, and offensive odor initially increased as additional chemical indicators were introduced and subsequently approached a plateau. Candidate models whose five-fold mean AUC values differed from the corresponding maximum value by no more than 0.01 were considered to have approximately optimal performance. Among the candidate models satisfying this criterion, the model containing the fewest chemical indicators was selected. Accordingly, 15, 14, and 15 key chemical indicators were ultimately selected for aroma quality, aroma intensity, and offensive odor, respectively, as presented in **Table 6**.

**Figure 3:**
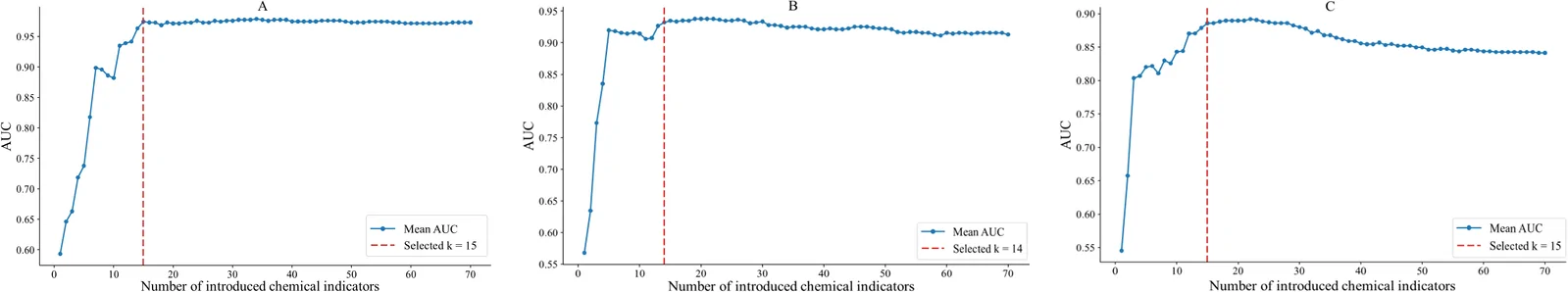
Changes in the mean five-fold cross-validation AUC with the incremental inclusion of chemical indicators: **(A)** aroma quality, **(B)** aroma intensity, and **(C)** offensive odor.

**Table 6:** Key chemical indicators for aroma quality, aroma intensity, and offensive odor.

Sensory indicator	Key chemical indicators
Aroma quality	Calcium, Vanillic acid, Magnesium, Malic acid, Chlorogenic acid, Dichloromethane extract, Phosphate, Fru-Tyr, Isoleucine, Palmitic acid, Glu-An, Chlorine, Succinic acid, Asparagine, and Leucine (15 chemical indicators)
Aroma intensity	Calcium, Magnesium, Malic acid, Myristic acid, Potassium, Sulfate, Total alkaloids, Palmitic acid, Vanillic acid, Chlorine, Neophytadiene, Dichloromethane extract, Glutamic acid, and Threonine (14 chemical indicators)
Offensive odor	Magnesium, Calcium, Chlorine, Malic acid, Aspartic acid, Vanillic acid, Total alkaloids, Oxalic acid, Fru-Tyr, Phosphate, Fru-Asn, Asparagine, Dichloromethane extract, Sulfate, and Threonine (15 chemical indicators)

The THT treatment temperature used in this study was 215 °C. Under this treatment condition, the contents of cations and anions in tobacco leaves, including calcium, magnesium, potassium, chloride, sulfate, and phosphate ions, remained essentially unchanged, making them unsuitable as effective targets for chemical regulation (Vassilev et al., 2010; Strezov et al., 2012; Olsson et al., 1997). Therefore, these six chemical indicators were further excluded from the key chemical indicators listed in **Table 6**. Shrinkage LDA binary classification models for aroma quality, aroma intensity, and offensive odor were then constructed using the remaining key chemical indicators and used as the decision models for counterfactual explanations and the targeted optimization of chemical indicators. Following this exclusion, 11, 9, and 10 remaining key chemical indicators were retained in the decision models for aroma quality, aroma intensity, and offensive odor, respectively, as shown in **Table 7**.

**Table 7:** Key chemical indicators included in the counterfactual decision models for aroma quality, aroma intensity, and offensive odor.

Sensory indicator	Key chemical indicators
Aroma quality	Vanillic acid, Malic acid, Chlorogenic acid, Dichloromethane extract, Fru-Tyr, Isoleucine, Palmitic acid, Glu-An, Succinic acid, Asparagine, and Leucine (11 chemical indicators)
Aroma intensity	Malic acid, Myristic acid, Total alkaloids, Palmitic acid, Vanillic acid, Neophytadiene, Dichloromethane extract, Glutamic acid, and Threonine (9 chemical indicators)
Offensive odor	Malic acid, Aspartic acid, Vanillic acid, Total alkaloids, Oxalic acid, Fru-Tyr, Fru-Asn, Asparagine, Dichloromethane extract, and Threonine (10 chemical indicators)

### 3.3 Construction and evaluation of decision models for counterfactual explanations

Decision models for counterfactual explanations of the corresponding sensory attributes were constructed using the selected key chemical indicators described above, and their classification performance on the independent test sets is presented in **Table 8**. The decision models for aroma quality, aroma intensity, and offensive odor all achieved accuracies above 84%, while their macro-averaged precision, macro-averaged recall, and macro-averaged F1 scores also exceeded 84%. These results indicate that all three decision models exhibited good classification performance and that good predictive mapping relationships existed between the remaining key chemical indicators and the corresponding sensory quality classes. On this basis, targeted regulation of the key chemical indicators may enable low-sensory-quality samples to cross the classification decision boundaries in the chemical feature space and enter the decision regions associated with the high-sensory-quality groups, thereby achieving targeted improvements in sensory quality.

**Table 8:** Performance of the models for aroma quality, aroma intensity, and offensive odor on the test set.

Sensory indicator	Accuracy	Precision	Recall	F1-score
Aroma quality	94.1%	94.0%	94.3%	94.1%
Aroma intensity	84.9%	85.1%	84.8%	84.9%
Offensive odor	87.5%	87.4%	87.6%	87.5%

### 3.4 Theoretical chemical-indicator regulation strategies for improving tobacco leaf sensory quality based on counterfactual explanations and their validation

Counterfactual explanations for sensory quality improvement were used to perform targeted optimization of the chemical indicators of the three control samples described in Section 2.1. Theoretical chemical-indicator adjustment strategies for improving each sensory attribute were obtained, and the corresponding optimized samples were determined. The results obtained for the experimental samples treated using the THT process (CL group) were used for validation. Sample YS-1 was selected as an example to illustrate the targeted improvement of aroma quality and aroma intensity.

#### 3.4.1 Chemical-indicator adjustment strategy for improving aroma quality

The adjustment strategy for the 11 key chemical indicators of validation sample YS-1 is presented in **Table 9**. All 11 indicators required indicator-specific adjustments. Among them, Fru-Tyr, vanillic acid, malic acid, chlorogenic acid, and isoleucine required increases of varying magnitudes, ranging from 0.697% to 9.34%. In contrast, dichloromethane extract, asparagine, leucine, Glu-An, palmitic acid, and succinic acid required decreases of varying magnitudes, ranging from 0.715% to 37.6%.

**Table 9:** Chemical indicator regulation scheme for improving the aroma quality of YS-1.

Chemical indicator	YS-1	Regulation target	Change (%)
Dichloromethane extract (%)	5.05	4.66	−7.69
Asparagine (μg/g)	997	622	−37.6
Leucine (μg/g)	15.1	13.6	−9.95
Fru-Tyr (μg/g)	83	90.8	9.34
Glu-An (μg/g)	108	68.5	−36.6
Vanillic acid (mg/g)	0.112	0.117	4.17
Malic acid (mg/g)	61	66.5	9.06
Chlorogenic acid (mg/g)	7.22	7.75	7.28
Palmitic acid (mg/g)	3.23	3.21	−0.715
Succinic acid (mg/g)	0.252	0.242	−3.79
Isoleucine (μg/g)	10.8	10.9	0.697

Percentage changes were calculated using unrounded values.

Through the targeted adjustment of these 11 chemical indicators, the theoretical discriminant score for aroma quality was successfully increased to the discriminant-score centroid of the high-sensory-quality group (3.06). Furthermore, the adjusted values of all 11 chemical indicators remained within the hard-bound constraints, indicating that the proposed adjustment strategy achieved the intended quality-improvement target while satisfying the predefined constraints.

Control sample YS-1 was treated using the THT process at 215 °C to obtain experimental sample CL-1. The changes in the chemical indicators of CL-1 relative to YS-1 are presented in **Table 10**. As shown in the table, among the 11 chemical indicators, eight indicators in CL-1, including dichloromethane extract, leucine, and Fru-Tyr, changed in the directions specified by the theoretical adjustment strategy, resulting in a directional agreement rate of 72.7%. Correspondingly, the discriminant score of the aroma quality decision model increased from −1.81 for validation sample YS-1 to 3.41 for CL-1, accompanied by an improvement in aroma quality. The eight indicators showing directional agreement, including dichloromethane extract, leucine, and Fru-Tyr, collectively increased the discriminant score of CL-1 relative to YS-1 by 6.01. In contrast, the three indicators showing inconsistent directions collectively decreased the discriminant score of CL-1 relative to YS-1 by 0.79. The combined effects of the indicators showing consistent and inconsistent directions resulted in a net increase of 5.22 in the discriminant score of the aroma quality decision model. These results indicate that the indicators showing directional agreement were the primary contributors to the increase in the aroma quality discriminant score, whereas the indicators showing inconsistent directions partially offset their positive contribution, with an offset magnitude of approximately 13.1%. Moreover, the sensory smoking evaluation results presented in **Table 1** showed that the aroma quality of CL-1 was superior to that of YS-1. Overall, the chemical-indicator adjustment strategy generated by the counterfactual explanations showed a high degree of agreement with the pattern of chemical-indicator changes induced by the processing treatment that improved the sensory aroma quality of YS-1.

**Table 10:** Comparison of chemical indicator changes between THT treatment and the theoretical regulation scheme.

Chemical indicator	YS-1	CL-1	THT-induced change (%)	Optimization-derived change (%)	Directional consistency
Dichloromethane extract (%)	5.05	4.67	−7.53	−7.69	Consistent
Asparagine (μg/g)	997	1,148	15.1	−37.6	Inconsistent
Leucine (μg/g)	15.1	14.6	−3.31	−9.95	Consistent
Fru-Tyr (μg/g)	83	87	4.82	9.34	Consistent
Glu-An (μg/g)	108	138	27.8	−36.6	Inconsistent
Vanillic acid (mg/g)	0.112	0.129	15.2	4.17	Consistent
Malic acid (mg/g)	61	63.2	3.61	9.06	Consistent
Chlorogenic acid (mg/g)	7.22	8.13	12.6	7.28	Consistent
Palmitic acid (mg/g)	3.23	2.86	−11.45	−0.715	Consistent
Succinic acid (mg/g)	0.252	0.245	−2.78	−3.79	Consistent
Isoleucine (μg/g)	10.8	9.84	−9.14	0.697	Inconsistent

#### 3.4.2 Chemical-indicator adjustment strategy for improving aroma intensity

The adjustment strategy for the nine chemical indicators of validation sample YS-1 is presented in **Table 11**. Of these nine indicators, seven required indicator-specific adjustments. Vanillic acid, neophytadiene, malic acid, total alkaloids, and threonine required increases of varying magnitudes, ranging from 1.04% to 8.44%. In contrast, myristic acid and dichloromethane extract required decreases ranging from 4.02% to 11.7%, whereas glutamic acid and palmitic acid required no adjustment.

**Table 11:** Chemical indicator regulation scheme for improving the aroma intensity of YS-1.

Chemical indicator	YS-1	Regulation target	Change (%)
Myristic acid (mg/g)	0.156	0.138	−11.7
Vanillic acid (mg/g)	0.112	0.121	8.44
Dichloromethane extract (%)	5.05	4.85	−4.02
Neophytadiene (mg/g)	0.747	0.763	2.14
Malic acid (mg/g)	61	62.4	2.28
Total alkaloids (%)	1.56	1.58	1.58
Threonine (μg/g)	77.2	78	1.04
Glutamic acid (μg/g)	134	134	–
Palmitic acid (mg/g)	3.23	3.23	–

Percentage changes were calculated using unrounded values.

Through the targeted adjustment of these nine chemical indicators, the theoretical discriminant score for aroma intensity was successfully increased to the discriminant-score centroid of the high-sensory-quality group (1.75). Furthermore, the adjusted values of all nine chemical indicators remained within the hard-bound constraints, indicating that the proposed chemical-indicator adjustment strategy also achieved the intended quality-improvement target while satisfying the predefined constraints.

Control sample YS-1 was treated using the THT process at 215 °C to obtain experimental sample CL-1. The changes in the chemical indicators of CL-1 relative to YS-1 are presented in **Table 12**. As shown in the table, among the nine chemical indicators, six indicators in CL-1-myristic acid, vanillic acid, dichloromethane extract, neophytadiene, malic acid, and total alkaloids-changed in the directions specified by the theoretical adjustment strategy, resulting in a directional agreement rate of 66.7%. Correspondingly, the discriminant score of the aroma intensity decision model increased from −0.79 for validation sample YS-1 to 2.52 for CL-1, accompanied by an improvement in aroma intensity. The six indicators showing directional agreement collectively increased the discriminant score of CL-1 relative to YS-1 by 2.80, whereas the three indicators showing inconsistent directions collectively increased the score by 0.51. The combined effects of the indicators showing consistent and inconsistent directions resulted in a net increase of 3.31 in the discriminant score of the aroma intensity decision model. The indicators showing directional agreement accounted for 84.6% of the total increase in the discriminant score and were therefore the primary contributors to the increase in the aroma intensity discriminant score of CL-1. Moreover, the sensory smoking evaluation results presented in **Table 1** showed that the aroma intensity of CL-1 was superior to that of YS-1. Overall, the chemical-indicator adjustment strategy generated by the counterfactual explanations showed a high degree of agreement with the pattern of chemical-indicator changes induced by the processing treatment that improved the sensory aroma intensity of YS-1.

**Table 12:** Comparison of chemical indicator changes between THT treatment and the theoretical regulation scheme for improving the aroma intensity of YS-1.

Chemical indicator	YS-1	CL-1	THT-induced change (%)	Optimization-derived change (%)	Directional consistency
Myristic acid (mg/g)	0.156	0.145	−7.05	−11.7	Consistent
Vanillic acid (mg/g)	0.112	0.129	15.2	8.44	Consistent
Dichloromethane extract (%)	5.05	4.67	−7.53	−4.02	Consistent
Neophytadiene (mg/g)	0.747	0.777	4.02	2.14	Consistent
Malic acid (mg/g)	61	63.2	3.61	2.28	Consistent
Total alkaloids (%)	1.56	1.6	2.56	1.58	Consistent
Threonine (μg/g)	77.2	69.3	−10.2	1.04	Inconsistent
Glutamic acid (μg/g)	134	102	−24.0	–	Inconsistent
Palmitic acid (mg/g)	3.23	2.86	−11.5	–	Inconsistent

#### 3.4.3 Validation results for the sensory attributes of samples YS-2 and YS-3

Targeted optimization was performed to improve the sensory attributes of validation samples YS-2 and YS-3. The numbers of chemical indicators adjusted in the corresponding sensory quality adjustment strategies are presented in **Table 13**. As shown in **Table 13**, nearly all chemical indicators included in the respective models required adjustment to achieve targeted improvements in aroma quality, aroma intensity, and offensive odor. These results are consistent with the established understanding that tobacco sensory quality is associated with the balance and interactions among multiple chemical components, rather than changes in a single component alone (Zhao et al., 2023).

**Table 13:** Summary of the theoretical chemical indicator regulation schemes for sensory quality improvement.

Sensory indicator	Theoretical regulation scheme	YS-2	YS-3
Aroma quality (11 chemical indicators)	Number of adjusted chemical indicators	11	11
	Number of increased chemical indicators and range of increase	5; 0.325%~7.33%	5; 0.307%~4.88%
	Number of decreased chemical indicators and range of decrease	6; 0.478%~20.4%	6; 0.386%~14.3%
Aroma intensity (9 chemical indicators)	Number of adjusted chemical indicators	9	8
	Number of increased chemical indicators and range of increase	6; 0.191%~5.69%	6; 0.420%~11.2%
	Number of decreased chemical indicators and range of decrease	3; 1.48%~8.06%	2; 0.722%~14.8%
Offensive odor (10 chemical indicators)	Number of adjusted chemical indicators	10	10
	Number of increased chemical indicators and range of increase	5; 1.07%~22.0%	5; 0.100%~8.97%
	Number of decreased chemical indicators and range of decrease	5; 1.00%~33.1%	5; 1.37%~23.4%

Control samples YS-2 and YS-3 were treated using the THT process at 215 °C to obtain experimental samples CL-2 and CL-3, respectively. According to the sensory quality scores presented in **Table 1**, the processing treatment resulted in improvements in the offensive odor of YS-2 and in the aroma quality and aroma intensity of YS-3.

The comprehensive evaluation of the changes in the offensive odor of YS-2 and the aroma quality and aroma intensity of YS-3 is presented in **Table 14**. As shown in **Table 14**, the directional agreement rates for the chemical indicators corresponding to the respective sensory attributes ranged from 66.7% to 72.7%. Moreover, for all three sensory attributes, the contributions of indicators showing directional agreement were consistently greater in magnitude than those of indicators showing inconsistent directions. The combined effects of these two groups of indicators therefore resulted in net increases in the model discriminant scores. These results further demonstrate that the chemical-indicator adjustment strategies generated by the counterfactual explanations showed a high degree of agreement with the patterns of chemical-indicator changes induced by the processing treatment that improved sensory quality.

**Table 14:** Comprehensive evaluation of sensory quality changes after THT treatment.

Sample	Sensory indicator	Directional consistency of chemical indicator changes		Effects on model discriminant score		
		Consistent indicators (n)	Consistency rate (%)	Consistent changes	Inconsistent changes	Combined effects
YS-2/CL-2	Offensive odor	7	70.0	1.41	-0.19	1.22
YS-3/CL-3	Aroma quality	8	72.7	5.34	-0.66	4.68
	Aroma intensity	6	66.7	1.81	-0.11	1.70

## 4 Conclusions

To address the challenge of targeted chemical-indicator regulation during the improvement of tobacco sensory quality, this study introduced an actionable counterfactual explanation mechanism. The main conclusions are as follows:

1. The key chemical indicators selected through chemical-indicator importance ranking and incremental feature selection maintained the classification performance and generalization ability of the machine-learning models while reducing feature dimensionality.

2. The theoretical chemical-indicator adjustment strategies showed good agreement with the actual patterns of chemical-indicator changes. During sensory quality improvement, the directional agreement rates between the actual changes in chemical indicators and the adjustment strategies generated by the counterfactual explanations ranged from 66.7% to 72.7%. Moreover,the contributions of indicators showing directional agreement were consistently greater in magnitude than those of indicators showing inconsistent directions, indicating that the indicators showing directional agreement were the primary contributors to improvements in the sensory quality of the samples.

It should be noted that the theoretically predicted adjustment magnitudes and the experimentally observed changes for some chemical indicators were relatively small and, in some cases, were close to the measurement errors associated with routine chemical analyses. Therefore, small changes in individual chemical indicators should be interpreted with caution. Nevertheless, across multiple validation results, the actual directions of chemical-indicator changes showed good agreement with the theoretically predicted adjustment pathways, providing preliminary experimental support for the validity of the counterfactual adjustment strategies. On the one hand, these findings are consistent with the understanding that tobacco sensory quality depends on the overall balance and synergistic effects of multiple chemical indicators. On the other hand, these findings provide preliminary evidence that the counterfactual explanation method for improving tobacco sensory quality used in this study has potential for exploring the directions of change in chemical indicators within complex chemical systems. Further experiments are needed to determine how this potential can be translated into practical application.

Supplementary evaluations of tobacco leaf samples from other production regions yielded similar results, suggesting that the proposed method may also be applicable beyond Yunnan Province. However, the results presented in this study focus on samples from Yunnan Province.

## 5 Limitation and future work

The method proposed in this study for improving tobacco leaves with low sensory quality based on counterfactual explanations provides clearly defined key chemical indicators and their adjustment magnitudes for shifting these tobacco leaves toward the high-quality class. However, quantitative relationships have not yet been established between changes in the contents of these key chemical indicators and tobacco-processing operations, such as toasting, aging, and casing, or their associated parameters, including temperature, processing duration, and heat-transfer conditions. To our knowledge, relevant studies on these relationships remain limited. Moreover, the practical optimization of tobacco leaves with low sensory quality must also consider industrial usability, process feasibility, treatment costs, and other relevant factors.

Future research could employ controlled experiments involving different combinations of processing parameters to systematically investigate the effects of processing conditions on key chemical indicators and sensory quality, thereby establishing quantitative relationships among processing parameters, chemical-indicator changes, and sensory-quality improvement. On this basis, factors such as the practically adjustable ranges of chemical indicators, process constraints, and treatment costs could be incorporated into the counterfactual optimization process. This would facilitate the translation of chemical-indicator adjustment targets generated through counterfactual explanations into operationally feasible quality-improvement strategies. Their application stability could subsequently be evaluated using different tobacco raw materials and independent processing batches.

## Data Availability

The raw data supporting the conclusions of this article will be made available by the authors, without undue reservation.

## References

[B1] An Y. Xu L. Yang J. Li B. Mao D. (2012). Effects of roasting conditions on major aroma components in Burley tobacco. Tobacco Sci. Technol. 45, 56–60, 84. doi: 10.3969/j.issn.1002-0861.2012.10.013

[B2] Bie R. Zhou T. Zhou X. Jiang B. Zhou Y. Qiu J. . (2022). Study on quality prediction model of Shandong tobacco based on XGBoost algorithm. Chin. Tobacco Sci. 43, 80–86, 93. doi: 10.13496/j.issn.1007-5119.2022.05.013

[B3] Blankertz B. Lemm S. Treder M. Haufe S. Müller K.-R. (2011). Single-trial analysis and classification of ERP components—a tutorial. NeuroImage 56, 814–825. doi: 10.1016/j.neuroimage.2010.06.048 20600976

[B4] Chen Z. Pang M. Zhao Z. Li S. Miao R. Zhang Y. . (2020). Feature selection may improve deep neural networks for the bioinformatics problems. Bioinformatics 36, 1542–1552. doi: 10.1093/bioinformatics/btz763 31591638

[B5] Cheng C. Yang S. Li H. Guan S. Yu C. Li G. . (2026). Effect of heavy casing addition and high-temperature baking on physicochemical properties and sensory quality of low-grade tobacco leaves in Shandong. J. Light Industry 41, 78–88. doi: 10.12187/2026.02.007 41709941 PMC12910233

[B6] Guidotti R. (2024). Counterfactual explanations and how to find them: Literature review and benchmarking. Data Min Knowl. Discov. 38, 2770–2824. doi: 10.1007/s10618-022-00831-630311153

[B7] Guo J. Zhao L. Liang Y. Wang D. Shang P. Li H. . (2023). Moisture-adaptive corrections of NIR for the rapid simultaneous analysis of 70 chemicals in tobacco: A case study on tobacco. Microchem J. 189, 108522. doi: 10.1016/j.microc.2023.10852242574925

[B8] Hao J. Ji Q. Li L. Zheng C. Wu N. Wu H. . (2022). Research progress on improving aroma of tobacco leaf with bio-enzymes and microorganism technology. Curr. Biotechnol. 12, 817–824. doi: 10.19586/j.2095-2341.2022.0070

[B9] Janisiów Ł. Kochańczyk M. Zieliński B. M. Danel T. (2026). Enhancing chemical explainability through counterfactual masking. Proc. AAAI Conf. Artif. Intell. 40, 22155–22163. doi: 10.1609/aaai.v40i26.39371

[B10] Korikov A. Beck J. C. (2021). “ Counterfactual explanations via inverse constraint programming”, in: 27th International Conference on Principles and Practice of Constraint Programming (CP 2021) (Dagstuhl: Schloss Dagstuhl-Leibniz—Zentrum für Informatik), 35:1–35:16. doi: 10.4230/LIPIcs.CP.2021.35

[B11] Li B. Li W. Guo J. Wang H. Wan R. Liu Y. . (2025). Outlier removal with weight penalization and aggregation: A robust variable selection method for enhancing near-infrared spectral analysis performance. Anal. Chem. 97, 7325–7332. doi: 10.1021/acs.analchem.4c07007 39970051

[B12] Liang M. Lei T. Zhan X. Liu S. Zhang X. Zou E. . (2025). Influence of torrefaction treatment on the pyrolysis and combustion characteristics of tobacco waste. J. Light Industry 40, 65–74. doi: 10.12187/2025.03.00841168888

[B13] Liang Y. Zhao L. Guo J. Wang H. Liu S. Wang L. . (2022). Just-in-time learning-integrated partial least-squares strategy for accurately predicting 71 chemical constituents in Chinese tobacco by near-infrared spectroscopy. ACS Omega 7, 38650–38659. doi: 10.1021/acsomega.2c04139 36340111 PMC9631892

[B14] Lin S. Zhang X. (2016). Correlation of tobacco chemical components and sensory quality assessed by PLSR. Chin. Tobacco Sci. 37, 78–82. doi: 10.13496/j.issn.1007-5119.2016.01.014

[B15] Liu H. Mao W. You M. Pan G. Guo C. Qiao X. . (2024). Optimization of biological fermentation for improving the quality of discarded tobacco leaves. Chin. Tobacco Sci. 45, 99–107. doi: 10.13496/j.issn.1007-5119.2024.02.012

[B16] Liu J. Gao H. Ma X. Yang Y. Wang X. Yang J. . (2016). Effect of high temperature baking on flue-cured tobacco chemical quality. J. Yunnan Agric. Univ. (Natural Science) 31, 880–885. doi: 10.16211/j.issn.1004-390X(n).2016.05.017

[B17] Ma Y. Gao X. Liu C. Li J. (2024). Improved SQP and SLSQP algorithms for feasible path-based process optimisation. Comput. Chem. Eng 188, 108751. doi: 10.1016/j.compchemeng.2024.10875142574925

[B18] Mothilal R. K. Sharma A. Tan C. (2020). “ Explaining machine learning classifiers through diverse counterfactual explanations”, in: Proceedings of the 2020 Conference on Fairness, Accountability, and Transparency (New York, NY: Association for Computing Machinery), 607–617. doi: 10.1145/3351095.3372850

[B19] Nie Z. (2025). “ Optimization for China’s industrial investment allocation”, in: Proceedings of the 2025 7th International Conference on Economic Management and Cultural Industry (ICEMCI 2025) (Paris: Atlantis Press), 67–74. doi: 10.2991/978-94-6463-888-2_9

[B20] Olsson J. G. Jäglid U. Pettersson J. B. C. Hald P. (1997). Alkali metal emission during pyrolysis of biomass. Energy Fuels 11, 779–784. doi: 10.1021/ef960096b

[B21] Ribeiro M. T. Singh S. Guestrin C. (2016). “ ‘Why should I trust you?’: Explaining the predictions of any classifier”, in: Proceedings of the 22nd ACM SIGKDD International Conference on Knowledge Discovery and Data Mining (New York, NY: Association for Computing Machinery), 1135–1144. doi: 10.1145/2939672.2939778

[B22] Schäfer J. Strimmer K. (2005). A shrinkage approach to large-scale covariance matrix estimation and implications for functional genomics. Stat. Appl. Genet. Mol. Biol. 4, Article 32. doi: 10.2202/1544-6115.1175 16646851

[B23] Shi H. (2011). Tobacco Flavor Chemistry (Beijing: China Agriculture Press).

[B24] Strezov V. Popovic E. Filkoski R. V. Shah P. Evans T. (2012). Assessment of the thermal processing behavior of tobacco waste. Energy Fuels 26, 5930–5935. doi: 10.1021/ef3006004

[B25] Su Z. Cao Y. Sun J. Hu Z. Zou K. Liu H. . (2025). Analysis of aroma differences in upper low-grade tobacco leaves pre- and post-bacterial-enzyme synergistic fermentation. J. Light Industry 40, 77–86. doi: 10.12187/2025.06.008

[B26] Vassilev S. V. Baxter D. Andersen L. K. Vassileva C. G. (2010). An overview of the chemical composition of biomass. Fuel 89, 913–933. doi: 10.1016/j.fuel.2009.10.02242574925

[B27] Verma S. Boonsanong V. Hoang M. A. Hines K. E. Dickerson J. P. Shah C. (2024). Counterfactual explanations and algorithmic recourses for machine learning: A review. ACM Comput. Surv 56, 1–42, Article 321. doi: 10.1145/3677119

[B28] Wachter S. Mittelstadt B. Russell C. (2018). Counterfactual explanations without opening the black box: Automated decisions and the GDPR. Harvard J. Law Technol. 31, 841–887. doi: 10.2139/ssrn.3063289

[B29] Wang S. (2018). Chemical composition analysis and prediction of tobacco. Chem. Eng. Trans. 71, 1009–1014. doi: 10.3303/CET1871169

[B30] Wellawatte G. P. Seshadri A. White A. D. (2022). Model-agnostic generation of counterfactual explanations for molecules. Chem. Sci. 13, 3697–3705. doi: 10.1039/D1SC05259D 35432902 PMC8966631

[B31] Xiao X. (1997). Tobacco Chemistry (Beijing: China Agricultural Science and Technology Press).

[B32] Xiong Z. Guan T. Zhou G. Ma R. Zhang M. Chen S. . (2023). Analysis of chemical and volatile components of tobacco leaves from Heilongjiang. Modern Agric. Sci. Technol., 184–187. doi: 10.3969/j.issn.1007-5739.2023.08.049

[B35] Zhao L. Shang S. Tian Y. Gao Y. Song Z. Peng L. . (2023). Integrative analysis of sensory evaluation and non-targeted metabolomics to unravel tobacco leaf metabolites associated with sensory quality of heated tobacco. Front. Plant Sci. 14, 1123100. doi: 10.3389/fpls.2023.1123100 36844088 PMC9944805

[B33] Zhou X. Li X. Liu Z. Zhou X. Qiu C. Zhuang Z. . (2024). Relationship between sensory quality and chemical component of flue-cured tobacco in Shandong Province and establishment of quality prediction models. Chin. Agric. Sci. Bull. 40, 128–134. doi: 10.11924/j.issn.1000-6850.casb2023-0579

[B34] Zhu Z. (2010). Improving usability of upper leaves, an important measure for accelerating up-grading cigarette quality. Tobacco Sci. Technol. 43, 5–9, 31. doi: 10.3969/j.issn.1002-0861.2010.06.001

